# Calibration of a Hyper-Spectral Imaging System Using a Low-Cost Reference

**DOI:** 10.3390/s21113738

**Published:** 2021-05-27

**Authors:** Muhammad Saad Shaikh, Keyvan Jaferzadeh, Benny Thörnberg, Johan Casselgren

**Affiliations:** 1 Department of Electronics Design, Mid Sweden University, 851 70 Sundsvall, Sweden; keyvan.jaferzadeh@mail.concordia.ca (K.J.); Benny.Thornberg@miun.se (B.T.); 2 Department of Computer Science and Software Engineering, Concordia University, Montreal, QC H3G 1M8, Canada; 3 Department of Engineering Sciences and Mathematics, Luleå University of Technology, 971 87 Luleå, Sweden; johan.casselgren@ltu.se

**Keywords:** hyperspectral imaging, push-broom camera, winter road conditions, calibration, teflon, spectralon, PTFE, dark current, InGaAs

## Abstract

In this paper, we present a hyper-spectral imaging system and practical calibration procedure using a low-cost calibration reference made of polytetrafluoroethylene. The imaging system includes a hyperspectral camera and an active source of illumination with a variable spectral distribution of intensity. The calibration reference is used to measure the relative reflectance of any material surface independent of the spectral distribution of light and camera sensitivity. Winter road conditions are taken as a test application, and several spectral images of snow, icy asphalt, dry asphalt, and wet asphalt were made at different exposure times using different illumination spectra. Graphs showing measured relative reflectance for different road conditions support the conclusion that measurements are independent of illumination. Principal component analysis of the acquired spectral data for road conditions shows well separated data clusters, demonstrating the system’s suitability for material classification.

## 1. Introduction

Spectral Imaging is a technique in which spectral information is shown as the third dimension of a two-dimensional spatial image (sometimes called a cube image). It was first proposed for remote sensing of Earth in 1985 [1]. Spectral imaging is popular in many scientific and engineering fields, including satellite-based remote sensing [2], agriculture [3], defense [4], medical diagnostics [5], food inspection [6], etc. In the listed applications, the spectrum of light emitted from a light source after having been diffusely reflected in the surface of various materials is analyzed. The illumination can be ambient light from the sun or an active source of illumination such as halogen lamps, xenon lamps, or LEDs. The reflection of light is a process that creates unique spectral signatures depending on the material composition of the reflecting surface [7]. Consequently, spectral imaging allows for the recording of unique spectral signatures in two dimensions such that the use of a trained classifier can determine the surface material at each individual pixel [8].

Spectral imaging is further divided into Multi-Spectral Imaging (MSI), Hyper-Spectral Imaging (HSI), and Ultra-Spectral Imaging. These abstractly defined divisions are based on spectral resolution, the number of spectral bands, and the width and gap between the bands. MSI generally collects spectral data in up to 10 different bands, which are generally non-contiguous, while HSI is able to collect data in hundreds of bands. Spectral imaging sensors collect information as a set of ‘images’. Each image represents a narrow wavelength range of the electromagnetic spectrum, also known as a spectral band. These images are combined to form a three-dimensional hyper-spectral data cube *I*(*x*, *y*, *λ*) for processing and analysis, were I represents intensity (or digital number), *x* and *y* represent two spatial dimensions of the scene, and *λ* represents the spectral dimension (comprising a range of wavelengths). The precision of these sensors is typically measured in spectral resolution, which is the width of each band of the spectrum that is captured.

**Figure 1:**
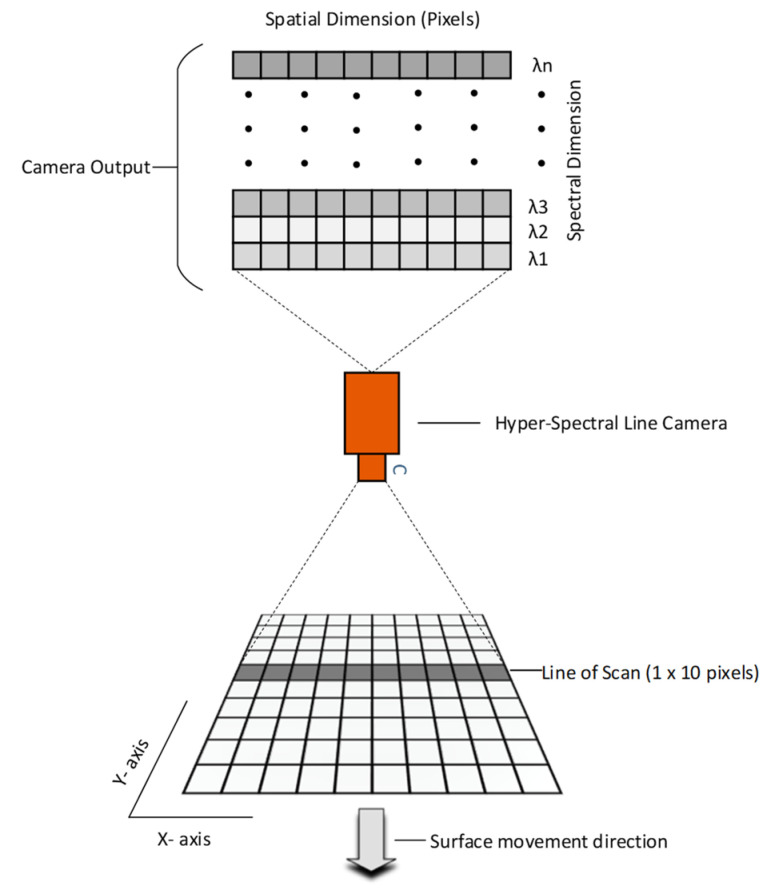
A general scheme for a push-broom camera. In this example, the spatial resolution of the camera is 10 pixels and the spectral resolution is n.

There are several different methods to acquire spectral image data. The most conventional techniques are the whisk-broom, push-broom, staring, and snapshot methods. We used the push-broom method in this research [9]. A push-broom camera scans a line of pixels on a surface at any given time and produces a two-dimensional output matrix *I*(*x*, *λ*), as depicted in Figure 1.

The center wavelengths *λ*_1_ to *λ_n_* for the spectral signals indexed by 1, …, *n*, shown in Figure 1, were defined by the camera manufacturer as the coefficients of a first order equation describing a straight line. The bandwidth of the same spectral signals was defined as a 8 nm full width half maximum. However, the sensitivities of those spectral signals were not defined and *I*(*x*, *λ*) is thus a digital number, without a unit, that captures the recorded dose of photons and dark current at a given exposure time. In addition, we need to consider the spectral distribution of the selected light source as unknown. Therefore, a method for calibration of the hyperspectral imaging system that is capable of suppressing variations in spectral distributions of light and sensitivities is needed [10,11,12,13,14,15,16,17,18].

Radiometric calibration of a hyperspectral camera means that the abstract digital numbers *I*(*x*, *λ*) that quantify the recorded outputs from each pixel and each spectral channel are given the concept of true radiation intensity [10] or true reflectance for a hyperspectral imaging system [15]. Calibration of true reflectance requires the use of one or more calibrated reflectance standards. Those calibration targets are typically made of a matte Lambertian reflecting surface, such that the reflecting light is of close to equal intensity in all directions. The total reflection integrated in all directions is typically close to 100 percent. Typically, a product called Spectralon^TM^ is used to manufacture reflectance standards [11,19]. Spectralon^TM^ is produced by pressurizing powder from polytetrafluoroethylene (PTFE) to form a block, followed by sintering it to bind the particles together. This results in an open-cell PTFE random matrix with approximately 40% void volume to enhance the scattering of light [19]. Teflon^TM^ is another more common trademark for a less expensive product made of solid PTFE. The price for a Spectralon^TM^ calibration standard is approximately 2.2 EUR/cm^2^ compared to 0.11 EUR/cm^2^ for a 10 mm thick board of solid PTFE.

In this paper, we argue that a calibration standard with an accurate reflectance close to 100 percent is not needed if the purpose is to identify materials by their spectral signatures. This is achievable because the spectral signatures can be recognized independently of the true scaling of reflectance 13. Similar arguments have been published by another research group [13], while other groups simply suggest using a white paper sheet for the calibration [17,18]. This paper defines the spectral calibration procedure by mathematical formulas. It is shown experimentally how variations in the spectral distribution of illumination can be efficiently suppressed after calibration using a solid PTFE board.

The basic mathematical formula for computation of calibrated true reflectance is defined in all referenced publications [11,12,13,14,16,17,18] as,

(1) $$\mathrm{reflectance}=100\times \frac{\mathrm{measurement}-\mathrm{dark}\;\mathrm{current}}{\mathrm{reference}-\mathrm{dark}\;\mathrm{current}}\;.$$

This formula implies that a measured image, a reference image, and a dark current image must be captured using a hyperspectral camera at the same exposure time. This is a consequence of the fact that the exposure time is simply omitted. Finding a common exposure time that is optimal for both the measured image and the reference can be a challenge. Therefore, it is shown in this paper how the concepts of intensity, dose, and exposure time extends the formulation of Equation (1) to allow different exposure times for reference and measurement.

In the next section, we present the details of the hardware, experimental setup, dark current analysis, data normalization, and the proposed calibration method, followed by the results and discussion. The paper ends with conclusions and references.

## 2. Materials and Methods

In this section, firstly we present details about the materials used in the experiments and the experimental setup used to generate the presented results. Secondly, the methods used for data acquisition, Dark Current (DC) modeling and estimation, and, finally, a formalization of the calibration procedure are presented.

### 2.1. Calibration Reference

An object made of a homogeneous well-defined material with well-defined optical properties to use as a reference for the spectral calibration of the camera is needed in combination with an illumination source. The requirements for this calibration reference are defined as:

(a) Diffused reflectance of light in the surface should be as evenly distributed as possible over the range of all wavelengths covered by the sensitivity of the spectral camera. In literature, this property is often referred to as ‘white reference’, which in the ideal case requires reflectance to be constant for all wavelengths.
(b) The material in the calibration reference should not absorb humidity, which would affect the reflectance. This is particularly important when performing calibrations in outdoor environments.
(c) The calibration object should be inexpensive, not more than 100 EUR for 850 cm^2^.

**Figure 2:**
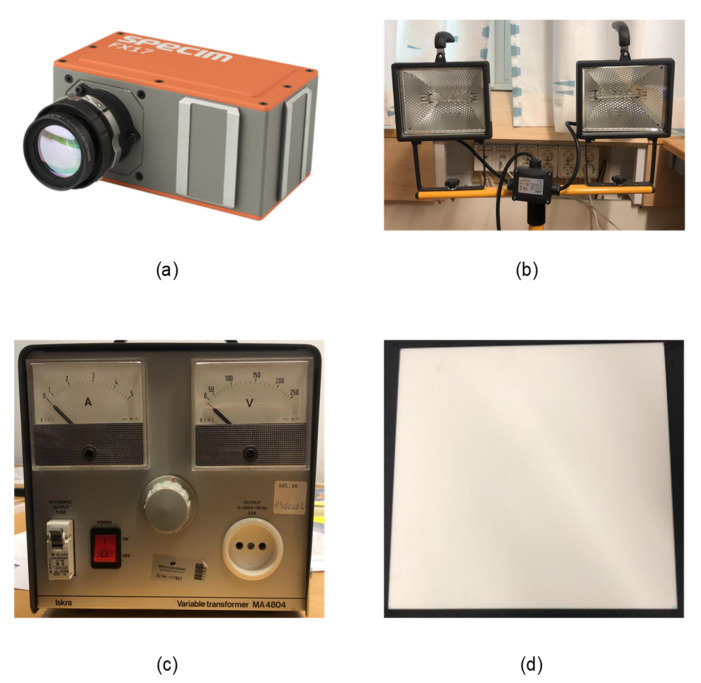
Materials used in the research: (**a**) FX17e Specim hyperspectral camera, (**b**) two halogen lamps mounted on a tripod, (**c**) variable transformer, and (**d**) PTFE tile.

A 10 mm thick, 295 × 295 mm polytetrafluoroethylene (PTFE) board manufactured by a Swiss company, Amsler and Frey (Schinznach-Dorf, Switzerland) was purchased. This board is shown in Figure 2d and was used as a calibration reference for all the experiments reported in this paper.

### 2.2. Hyperspectral Camera

**Figure 3:**
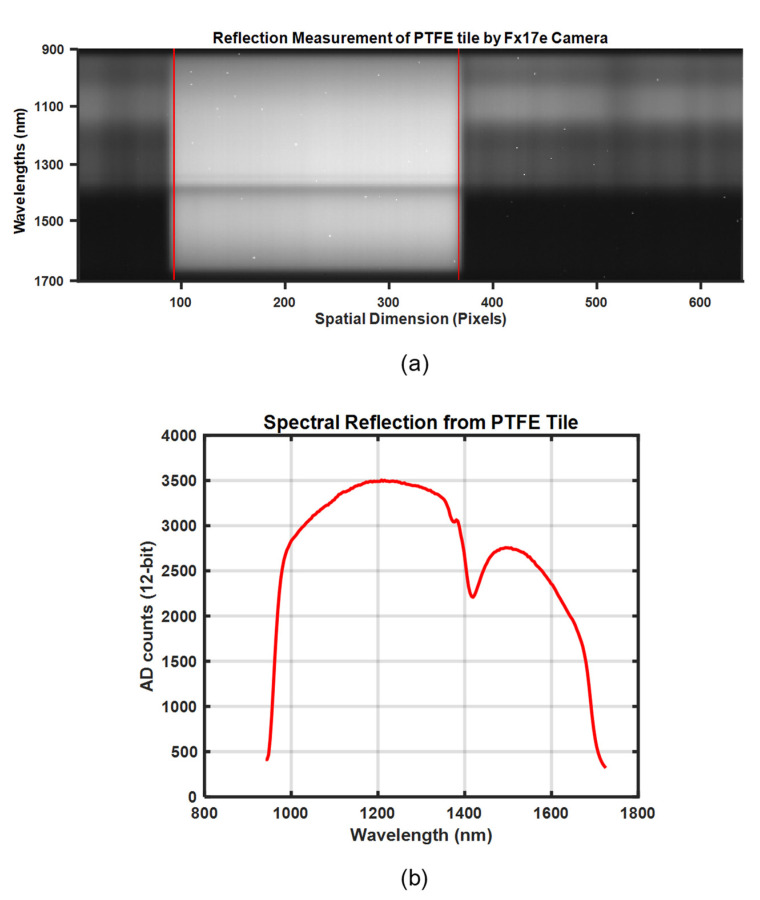
Camera FX17e output response for the PTFE tile: (**a**) unprocessed raw camera output and (**b**) average intensity of spectra for the area within two red lines.

In this research, a Specim FX17e hyper-spectral camera [20], as shown in Figure 2a, was used. This camera operates in the spectral range of 900–1700 nm and can capture 224 spectral bands with a full width half maximum (FWHM) of 8 nm. This is a line camera that uses the push-broom method⁠ to collect spectral information from single lines of 640 pixels, as illustrated in Figure 1. Motion, in combination with repeated spectral line captures, can be used to acquire a complete two-dimensional image in which the intensity of each pixel is distributed on 224 spectral bands. Figure 3a shows the raw unprocessed camera output of a PTFE surface where the horizontal and vertical axes represent the spatial *x*-dimension and spectral *λ*-dimension, as shown in Figure 1. The region of interest (ROI) between the two red lines in Figure 3a encompasses the area between columns 97 and 375. Throughout this research, the average of manually defined ROIs to measure the spectra of light reflected in the preferred calibration reference was used, as shown in Figure 3b. It could also be seen that signals at the longest and shortest wavelengths are weak. Therefore, we excluded the 12 uppermost and lowermost bands from the waveforms for every measurement in this work. This pruning is a result of the low intensity of long and short wavelengths in the FX17 camera, as specified in the datasheet [20]. This camera has an electrically controlled mechanical shutter that typically is used to capture dark current profiles; for further details, see Section 2.5. F-number is F/1.7, the field of view (FOV) is 18° and peak signal to noise ratio (PSNR) is 1000:1. Also, the camera can be utilized in 8-bit or 12-bit reading modes, and 12-bit was used for the spectral reading discussed in this paper.

### 2.3. Source of Active Illumination

**Figure 4:**
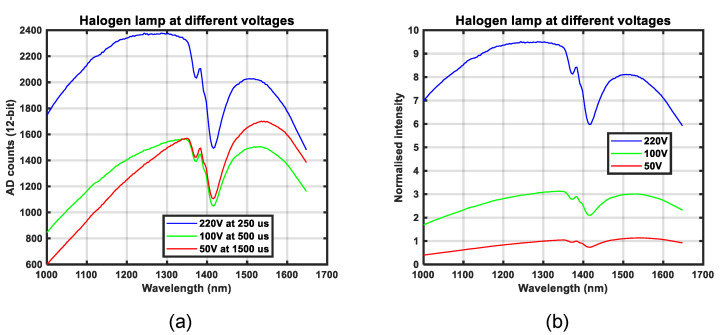
Camera response for the spectral distribution of halogen light at different lamp voltages: (**a**) Raw data output from the camera has the concept of a light dose without a unit, (**b**) Normalized intensity without a unit.

**Figure 5:**
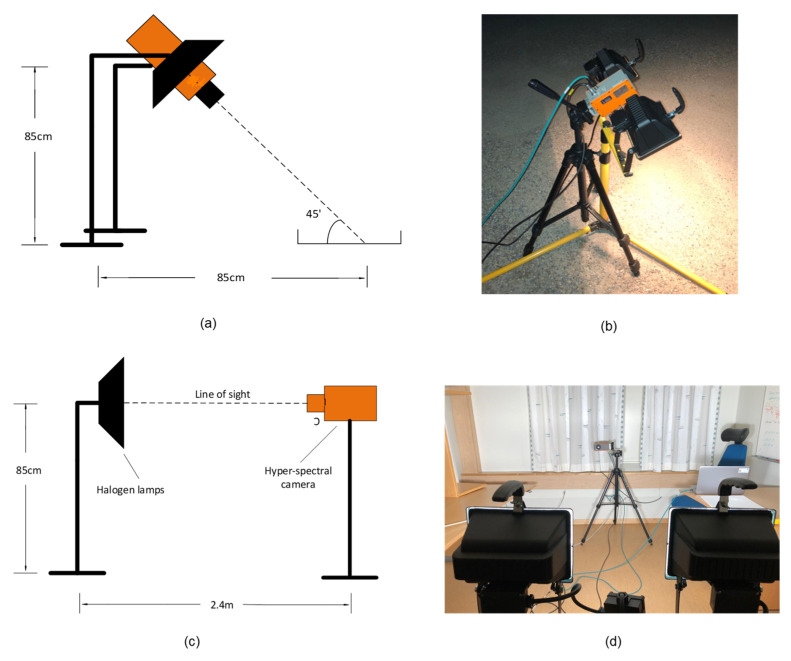
Experiment setup: (**a**) schematic depiction of the experiment setup, (**b**) photo of experiment setup, (**c**) schematic depiction of direct reference measurements, and (**d**) photo of direct reference measurements.

To illuminate the surfaces during our experiments, two halogen lamps, shown in Figure 2b, were used. The lamps were connected to a power source through a variable voltage transformer, shown in Figure 2c. Adjustments of the voltage level from 0 V to 220 V resulted in a variation in both the average intensity and spectral distribution of the emitted light. Figure 4 shows the spectral distribution of the halogen illumination at three different lamp voltages: 50 V, 100 V, and 220 V. Figure 4a shows the concept of light dose as the raw camera output at three different exposure times, while Figure 4b shows the concept of normalized light intensity. This normalization is explained in Section 2.6. It should be noted that the presented spectral distributions are the result of the combination of the spectral distribution of the camera sensitivity and light, which is further explained in Section 2.6. The data in Figure 4 were acquired by aiming the camera directly towards the illumination source, as depicted in Figure 5c. Intensities at longer wavelengths become stronger relatively at lower lamp voltages, since the temperature of the filament is expected to be lower. Higher voltages result in higher intensities at relatively shorter wavelengths. The variation in spectral distribution can be explained by the theory of black body radiation [21]. This variation in illumination properties was used to support our experiments on spectral calibration.

### 2.4. Experimental Setup

All data were acquired using the same experimental setup, depicted in Figure 5a,b. We positioned the camera between the two halogen lamps, at a height of 85 cm, all facing downwards at an angle of approximately 45 degrees relative to the ground plane. This angle was selected to minimize specular reflections from the analyzed surface while still getting a strong enough signal from the diffused reflections. The same experimental setup was used for the acquisition of all readings of the calibration reference and different conditions of asphalt. The readings were recorded at temperatures between −2 °C and −6 °C (outside conditions) in darkness at night. The stray light from distant streetlamps could have added a small amount of background bias to the measurements. However, the only dominant sources of illumination at the time of the experiments were the halogen lamps included in the experimental setup. Figure 5c,d shows the experimental setup used for direct measurements of the spectral distribution of the halogen light. The distance between the camera and lamps in Figure 5c was chosen to be approximately equal to the traveled path of light in air, as depicted in Figure 5a.

### 2.5. Dark Current Modeling and Estimation

**Figure 6:**
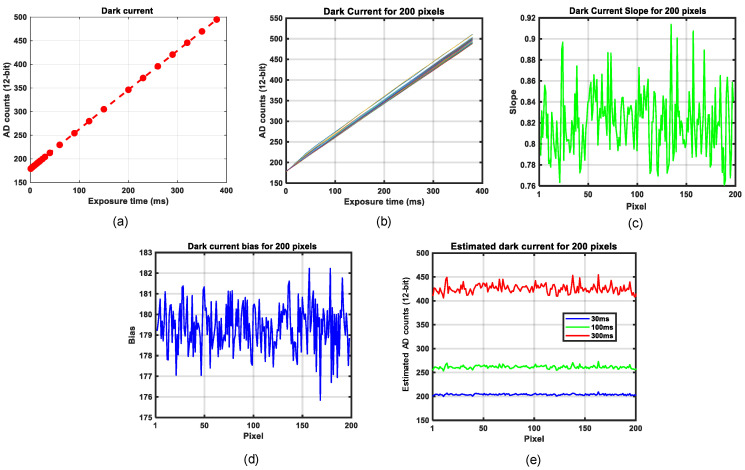
Dark current analysis. (**a**) Mean dark current values versus exposure time; (**b**) Dark current versus exposure time for 200 pixels; (**c**) Slope of dark current for 200 pixels; (**d**) Bias of dark current for 200 pixels; (**e**) Dark current estimation plots for 200 pixels at three different exposure times.

Most photo-detectors produce a signal current proportional to the incident optical power. However, even in the absence of any light input, there is still a very small amount of current present, known as dark current [22]⁠. It is a thermal phenomenon resulting from electrons that are spontaneously generated within the detector chip (valence electrons are thermally excited into the conduction band). Although independent of the signal level, it is dependent on the temperature of the sensor as well as the exposure time [23]⁠⁠. It is essential to understand how dark current relates to the exposure time for the hyperspectral camera used in this research. We acquired several images at different exposure times and plotted the mean values of pixel intensities. It turned out that the dark current increases linearly with the exposure time. The dependency of exposure time is shown in Figure 6a. Thus, the dark current *DC*(*t*) can be modeled using exposure time *t* and two coefficients for a line equation: bias and slope,

(2) DC(t)=bias+slope·t .

However, there is variation in every pixel’s response to dark current, as shown in Figure 6b. The corresponding variation of bias and slope are plotted in Figure 6c,d. A dark current model can be used to estimate the dark current at any exposure time during spectral data acquisition. Figure 6e shows examples of estimated dark current at 30, 100, and 300 ms. The benefit is that it is no longer necessary to measure the dark current for every single spectral measurement. Instead, the dark current response of the camera is modeled once at a specified ambient temperature and then a figure is estimated from this. This method assumes that the dark current measurements are independent of ambient temperature, which is not true for an InGaAs detector used in the NIR spectrum. However, the Fx17 camera from Specim has a built-in Peltier cooler that assures low chip temperatures, even at high ambient temperatures.

**Figure 7:**
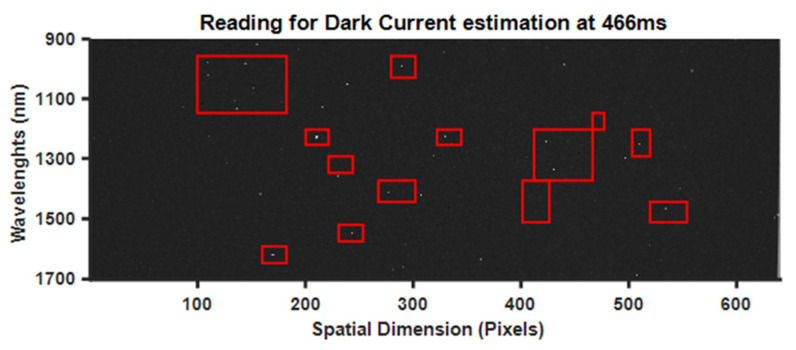
Hot pixels processing—image was taken with a closed shutter at the maximum exposure time (466 milliseconds). Red rectangles enclose regions with some of the more dominant clusters of hot pixels.

Apart from the dark current, star-like pixels, as shown in Figure 7, can be observed too. These are known as hot pixels; they have a very high excitement rate at the exposure time and therefore cannot provide reliable information. The effects of these pixels were reduced by applying a 5 × 5 pixel median filter. Median filtering is a simple, widely used non-linear rank-order filter that is capable of attenuating the accompanying noise from an image while preserving its details.

### 2.6. Normalization of Camera’s Raw Output

The camera output readings are mainly proportional to a dose of photon energy, converted into electrical charges, accumulated in capacitors adjacent to the sensitive pixel areas. A minor part of the output stems from a dose of dark current *DC*(*t*)*,* as explained in Section 2.5. Let *L*(*λ*) represent the spectral distribution of light intensity for the illumination source. Let *C*(*λ*) represent the spectral distribution of sensitivity for the camera. We modeled the camera output *D*(*λ*, *t*) for each spectral channel *λ* as,

(3) D(λ,t)=L(λ)·C(λ)·t+DC(t).

**Figure 8:**
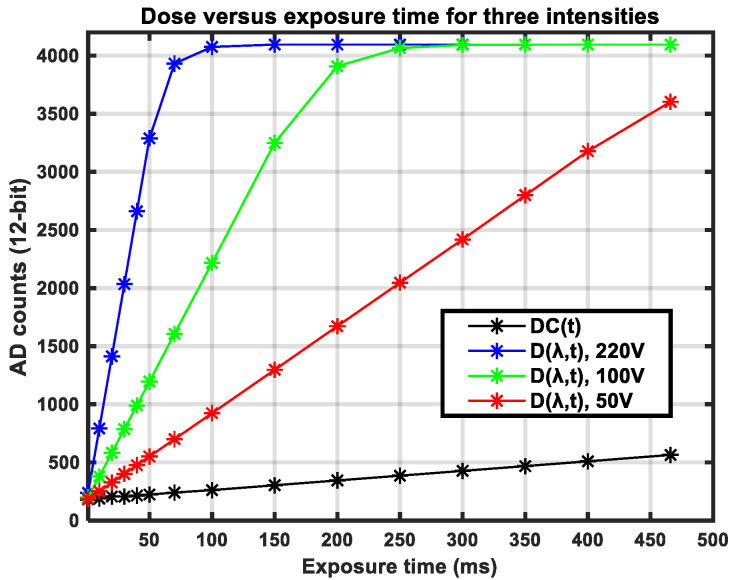
Dose of light and dark current for three different intensities.

The camera output *D*(*λ*, *t*) is thus a combined dose of light and dark current. Figure 8 shows the raw hyperspectral camera output from measurements of light reflected in the surface of the PTFE tile shown in Figure 2d. The dose *D*(*λ*, *t*) is increasing close to linearly with exposure time, except for values very close to saturation. An additional curve for dark current *DC*(*t*) is included.

A normalized measurement of light *R*(*λ*) that is independent of the exposure time *t* and dark current is essential such that it carries the concept of light intensity,

(4) $$R\left(\lambda \right)=\frac{D\left(\lambda ,t\right)-DC\left(t\right)}{t}=L\left(\lambda \right)\cdot C\left(\lambda \right).$$

The measurement *R*(*λ*) is still dependent on the spectral distribution of camera sensitivity *C*(*λ*)*,* which we consider during the spectral calibration.

### 2.7. Spectral Calibration

The experimental setup presented in Section 2.4 includes a spectral camera and illumination source. The sensitivity of the spectral bands of this camera is considered to be unknown. The spectral distribution of light intensity for the source is also considered to be unknown. Therefore, a calibration procedure is needed to measure the relative reflectance of the analyzed surface independently of different combinations of cameras and light sources. It should be noted that the absolute radiometric calibration is not the scope of this study.

**Definition** **1.** *The relative reflectance is defined as the wavelength-dependent fraction of reflected light intensity from the analyzed surface versus the reflected light intensity from the calibration reference*.

The calibration procedure proposed in this paper requires the use of the calibration reference presented in Section 2.1. Equations describing the computations executed at calibration are now developed.

Let W(λ) represent the spectral distribution of reflectance for the calibration reference. *M*(*λ*) represents the material-dependent spectral distribution of reflectance of the surface being analyzed. Thus, an expression for the measurement of light reflected in the surface of the calibration reference can be developed by:

(5) WR(λ)=L(λ) W(λ) C(λ).

Equally, the measurement of light reflected in the surface of the unknown material being analyzed can be shown by:

(6) MR(λ)=L(λ) M(λ) C(λ).

Both *WR*(*λ*) and *MR*(*λ*) are measurements taken using the hyperspectral camera, and the corresponding camera outputs were therefore normalized using the method described in Section 2.6. The relative reflectance in the analyzed surface could then be computed as,

(7) $$RR\left(\lambda \right)=\frac{MR\left(\lambda \right)}{WR\left(\lambda \right)}=\frac{L\left(\lambda \right)\;M\left(\lambda \right)\;C\left(\lambda \right)}{L\left(\lambda \right)\;W\left(\lambda \right)\;C\left(\lambda \right)}=\frac{M\left(\lambda \right)}{W\left(\lambda \right)}\;.$$

We could then conclude that the computed relative reflectance *RR*(*λ*) is independent of the illumination source *L*(*λ*) and camera *C*(*λ*). Finally, the material-dependent reflectance *M*(*λ*) could be computed if we knew the spectral distribution of reflectance for the calibration reference W(λ),

(8) M(λ)=W(λ)·RR(λ) .

Without knowing W(λ), someone can assume that the chosen calibration reference satisfies the conditions for an ‘ideal white reference’, as explained in Section 2.1. We simply said W(λ)=k*,* where k is the unknown reflectance of the calibration reference, which is constant for all wavelengths. Another option is to find the true value of *W*(*λ*) by measuring it. In this case, an additional measurement of the spectral distribution of light intensity was recorded with the camera directly facing the illumination source,

(9) DL(λ)=s·L(λ)·C(λ).

Typically, a much higher intensity will be recorded when the camera is facing towards the filament of the illumination source; compare Figure 5a,c. This intensity scaling is modeled using the constant *s*. The combination of Equations (5) and (9) allowed us to compute the spectral distribution of reflectance for the calibration reference,

(10) $$\frac{WR\left(\lambda \right)}{DL\left(\lambda \right)}=\frac{L\left(\lambda \right)\;W\left(\lambda \right)\;C\left(\lambda \right)}{s\cdot L\left(\lambda \right)\;C\left(\lambda \right)\;}=\frac{1}{s}\cdot W\left(\lambda \right)\;.$$

This characterization of the calibration reference *W*(*λ*) could then be used in Equation (8) to compute the material-dependent spectral distribution of reflectance *M*(*λ*)*,* thus making it completely independent of the chosen combination of the calibration target, camera, and illumination source.

### 2.8. Data Acquisition

**Figure 9:**
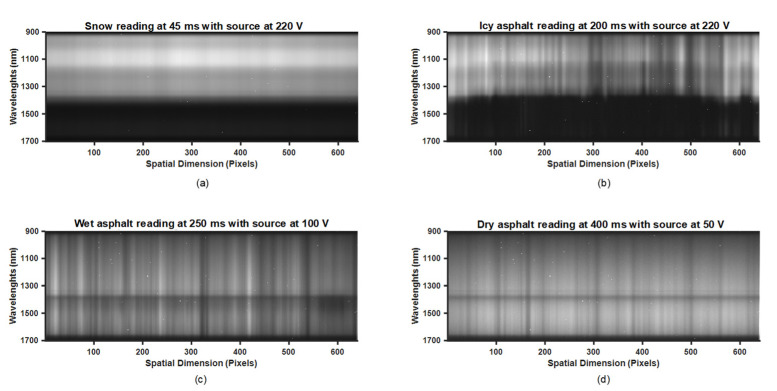
Camera’s actual output response for different road conditions: (**a**) snow, (**b**) icy, (**c**) wet, and (**d**) dry.

Spectral measurements of light reflected in an asphalt surface were recorded for three different combinations of exposure times and lamp voltages. This procedure was repeated for all asphalt conditions, each at three different locations. For each location, we moved the experimental setup and conducted new spectral measurements of the calibration reference for the three different lamp voltage levels of 50 V, 100 V, and 220 V. Eventually, direct spectral measurement of the illumination source by facing the source directly towards the camera was conducted, as can be seen in Figure 5c,d. The purpose of these measurements was to characterize the combination of the camera and illumination source. During data acquisition, we made sure that none of the pixel values reached the saturation level of the 12 bits dynamic range. Figure 9 shows the raw spectral data from snow, icy asphalt, wet asphalt, and dry asphalt at exposure times of 45 ms, 200 ms, 250 ms, and 400 ms, respectively. As illustrated in Figure 1, every row in these images represents a line of intensities in one of the 224 total spectral bands. For the acquisition of spectral data using the push-broom camera, a customized software was developed that runs on a Linux platform. This software has a GUI, developed using Qtcreator IDE, for a more user-friendly experience. OpenCV is used for the presentation and storage of image and video data.

### 2.9. Principal Component Analysis

Principal Component Analysis (PCA) is a method for linear projection of multidimensional data onto a new set of orthogonal vectors called Principal Components (PC). The data vectors after projection are referred to as scores, and the cosines of angles defining the PC are called loadings. The projection was chosen to minimize the correlation between the dimensions of scores. When the original data vectors show a high degree of correlation between their dimensions, only a few PC are needed to describe most of the data variance. PCA is a statistical method frequently used for, e.g., band selection in the field of hyperspectral imaging [8,24]. It is also widely used for dimensionality reduction in the visualization of hyperspectral imaging and classification studies in applications suffering from the curse of dimensionality.

### 2.10. Support Vector Machines (SVMs)

A support vector machine (SVM) is a supervised learning model with an associated learning algorithm that is widely used for data classification and regression analysis. In this work, we trained two SVM classifiers using MATLAB multiclass error-correcting output codes (ECOC) [25], to classify non-calibrated raw data and calibrated data, respectively in four classes to understand whether the proposed calibration method improves the classification. The classifiers were trained using radial base function (RBF) kernels. A total of 90% of data was used for training and 10% was used as test data. The data were randomly shuffled before separating test data.

## 3. Results and Analysis

**Figure 10:**
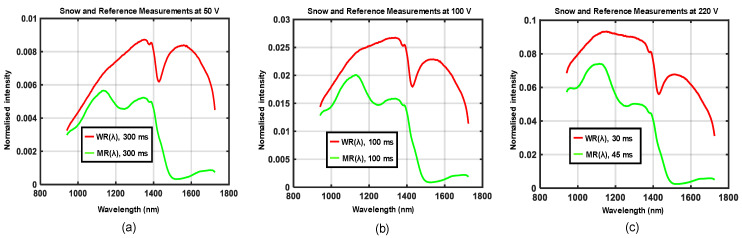
Spectral distributions of light reflected in snow *MR*(*λ*), the calibration reference *WR*(*λ*) and for three different voltages: (**a**) 50 V, (**b**) 100 V, and (**c**) 220 V.

**Figure 11:**
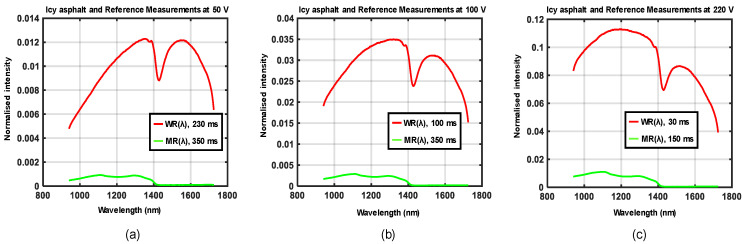
Spectral distributions of light reflected in icy asphalt *MR*(*λ*), in the calibration reference *WR*(*λ*), and for three different voltages: (**a**) 50 V, (**b**) 100 V, and (**c**) 220 V.

**Figure 12:**
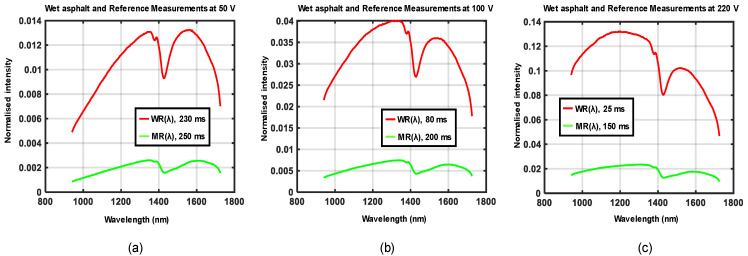
Spectral distributions of light reflected in wet asphalt *MR*(*λ*), the calibration reference *WR*(*λ*), and for three different voltages: (**a**) 50 V, (**b**) 100 V, and (**c**) 220 V.

**Figure 13:**
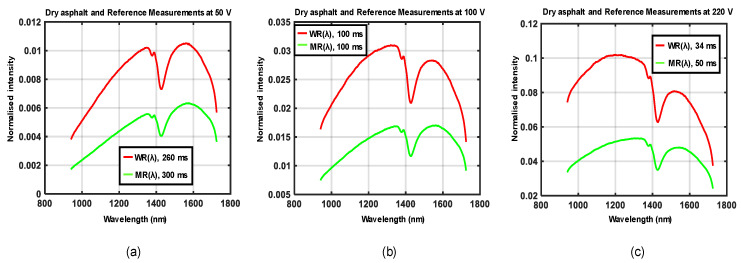
Spectral distributions of light reflected in dry asphalt *MR*(*λ*), the calibration reference *WR*(*λ*), and for three different voltages: (**a**) 50 V, (**b**) 100 V, and (**c**) 220 V.

The experimental setup shown in Figure 5a,b was used to acquire data for four different road conditions, each at three different locations. Three different supply voltages were generated by the variable transformer at each location such that the emitted halogen light showed a variation in spectral distributions. Waveforms in Figure 10, Figure 11, Figure 12 and Figure 13 show the spectral distributions of reflected light *MR*(*λ*) for the different road conditions: snow, icy asphalt, dry asphalt and wet asphalt, and three different voltage levels. Spectral distributions of light reflected in the surface of the calibration reference *WR*(*λ*) were measured at the same location as the corresponding road condition without moving the experimental setup. It is obvious that the higher lamp voltage results in greater illumination intensity for shorter wavelengths. A low lamp voltage gives greater intensity to longer wavelengths. Different exposure times were used for the measurement of analyzed surface *MR*(*λ*) and for the reference *WR*(*λ*), as indicated in Figure 10, Figure 11, Figure 12 and Figure 13. Therefore, *WR*(*λ*) and *MR*(*λ*) were normalized as described in Section 2.6 and the corresponding model is described by Equations (3), (4) and (6).

**Figure 14:**
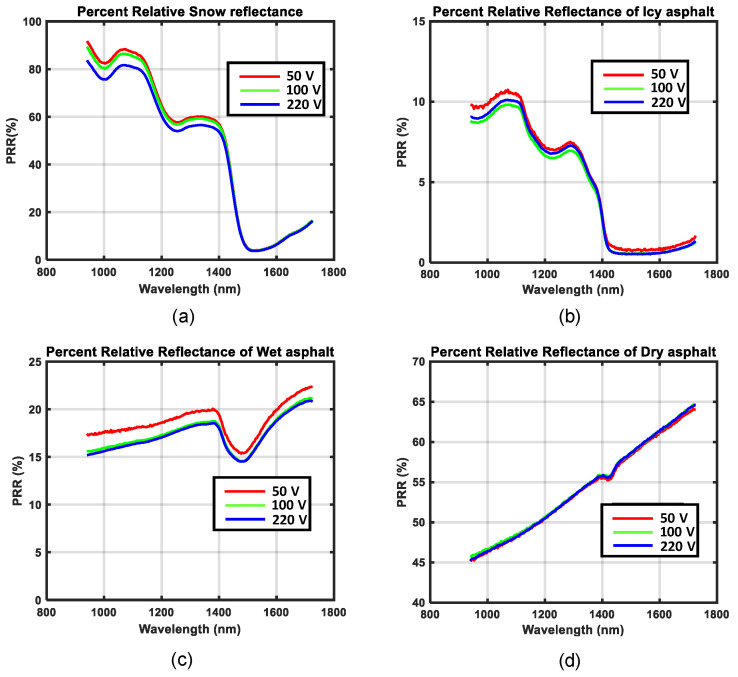
Relative reflectance *RR*(*λ*) of different road conditions as a percentage *PRR*(*λ*): (**a**) snow, (**b**) icy asphalt, (**c**) wet asphalt, and (**d**) dry asphalt.

The measurements of spectral distributions of reflected light, *MR*(*λ*) and *WR*(*λ*)*,* shown in Figure 10, Figure 11, Figure 12 and Figure 13, are dependent on both the illumination source and camera sensitivity, as shown by Equations (5) and (6). In order to eliminate these unwanted dependencies, the relative reflectance *RR*(*λ*) was calculated (as a percentage; see Figure 14). Figure 14 shows separate graphs for the different road conditions: snow, icy asphalt, dry asphalt and wet asphalt. There was a high degree of similarity between the curves corresponding to the three different lamp voltages, which verified the desired independence of illumination. There was also a lot of similarity with published spectral profiles of snow [26] when compared with Figure 14a. Similar absorption peaks could be observed at approximately 1.0, 1.2, and 1.5 µm for snowy asphalt and published reflectance for snow. For icy asphalt, we could recognize similar absorption peaks at 0.9 and 1.2 µm. When comparing wet asphalt in Figure 14c with dry asphalt in Figure 14d, we noted an absorption peak at approximately 1.5 µm that comes from the water molecule.

**Figure 15:**
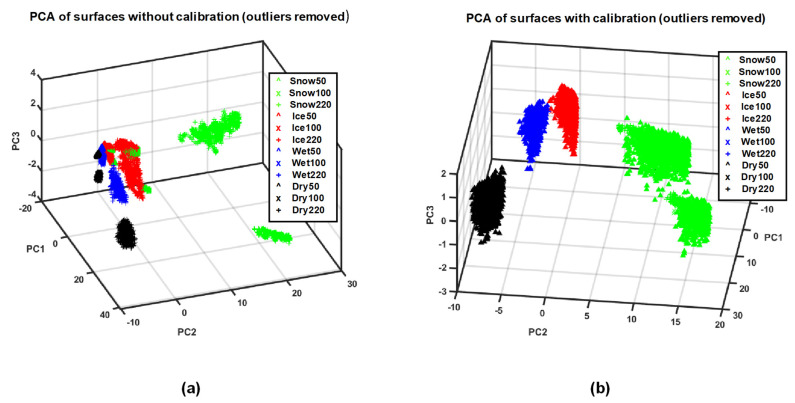
PCA plot of data vectors (**a**) uncalibrated spectral data *MR*(*λ*) (**b**) calibrated spectral data *RR*(*λ*).

PCA of both calibrated data vectors *RR*(*λ*) and non-calibrated raw data vectors *MR*(*λ*) for all of the road conditions were measured. Data vectors recorded at different illumination spectra for all four classes were labeled according to the road conditions: snow, icy asphalt, dry asphalt, and wet asphalt. The score plots of PCAs are presented in Figure 15. Data vectors of calibrated data *RR*(*λ*) for the different road conditions are concentrated into well-separated clusters (Figure 15b), compared to that of non-calibrated data *MR*(*λ*) (Figure 15a). Furthermore, our PCA analysis revealed that the three principal components describe more than 98% of the variance of the *RR*(*λ*) data analyzed for the calibrated data.

Since a hyperspectral reading provides a feature vector equal to the number of bands, PCA can be used to reduce the dimensions. Thus, the dimension of all score vectors was reduced after utilizing the PCA, then score data was used for training two SVM classifiers.

Table 1 shows the miss-classification rate of both classifiers trained with different numbers of PCs.

**Figure 16:**
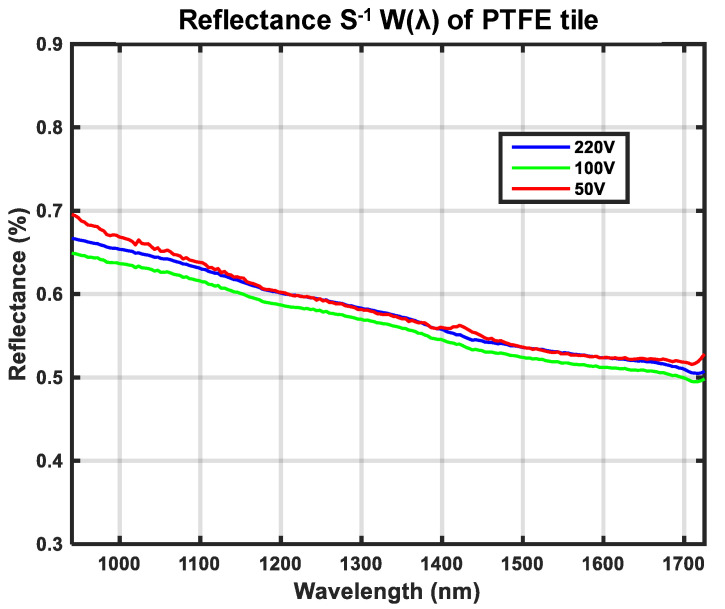
Spectral distribution of reflectance *W*(*λ*) for the calibration reference.

The spectral distribution of reflectance for the calibration reference *W*(*λ*) is shown in Figure 16. The close similarity of the illumination spectra generated from lamp voltages 50 V, 100 V, and 220 V verify that the computed *W*(*λ*) is close to being independent of the illumination source. Ideally, *W*(*λ*) should only be dependent on the material chosen for the calibration reference and independent of the illumination source and camera. The computation of *W*(*λ*) using Equation (10), therefore requires the spectral distribution of light intensity DL(λ) to be measured separately using the same camera; for more details, see Equation (9). Figure 5c depicts how the camera was directed towards the light source. The recorded light intensities are much higher for direct measurements in accordance with Figure 5c than compared to the setup in Figure 5a. This unknown scaling of intensities was modeled with the constant *s*; see Equation (9) for further details, which also explains the low reflectance values in Figure 16.

**Figure 17:**
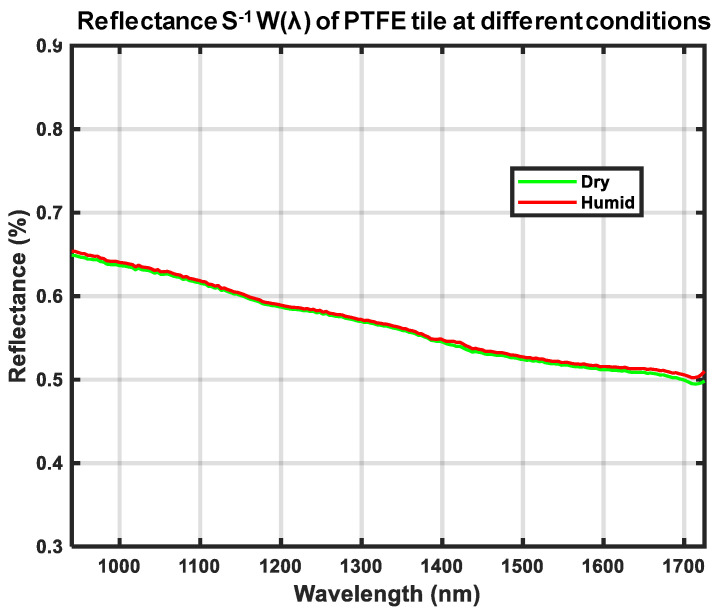
Spectral distribution of reflectance *W*(*λ*) for the calibration reference in humid and dry conditions. Measurements were made using a 100 V lamp voltage.

Figure 17 shows the computed *W*(*λ*) from a similar measurement of our calibration reference that was first submerged in water for 24 h. We wiped the visible water from the surface of the calibration reference and then measured *WR*(*λ*). The calibration reference was left to dry in an oven for 2 h at 70 °C and then the same measurement of *WR*(*λ*) was made again. The computed *W*(*λ*) for dry and humid conditions in Figure 17 are close to identical, a requirement defined in Section 2.1.

## 4. Discussion

The purpose of this research was to develop a method of spectral imaging to measure the relative reflectance *RR*(*λ*) of various surfaces, independent of the spectral distribution of both the light intensity of the illumination source *L*(*λ*) and the camera sensitivity *C*(*λ*). The analysis of road conditions was our test case. The relative reflectance of snow, icy asphalt, wet asphalt, and dry asphalt were successfully measured. These measurements were carried out using different exposure times for the calibration reference and for the analyzed surface. The results from these measurements, presented in Figure 14, match closely with the known signatures of snow, ice, and wet asphalt presented in previous studies [26,27]. Therefore, the calibration procedure required to compute *RR*(*λ*) can be considered as feasible, assuming that the calibration reference is close to a ‘white reference’, as explained in Section 2.1.

More accurate measurements of the spectral distribution of reflectance for a material *M*(*λ*) require a more advanced calibration procedure to assure that the measured *M*(*λ*) is not only independent of the illumination source and the camera but also of the calibration reference. In theory, it is shown that this is possible using Equation (8) if the spectral distribution of reflectance for the calibration reference *W*(*λ*) is known. For this reason, it is also shown how s^−1^*W*(*λ*) can be measured, as illustrated in Figure 16 and Figure 17. The constant *s* is an unknown scaling factor preventing true reflectance from being measured, while the relative spectral distribution of reflectance should still be correct. One of the referenced research groups argue that calibrated reflectance standards are not needed. They compared the use of three different non-standard materials for calibration: Acryllic, Cardboard, and Teflon [13].

Figure 16 shows that our PTFE calibration reference has similar reflectance to Spectralon^TM^ within a near-infrared light range, presented in [19]. Generally, the true reflectance of a material is measured using an apparatus that incorporates an integrating sphere [27]. We did not have access to an integrating sphere for this research. Instead, the experimental setups shown in Figure 5 were used to measure s^−1^*W*(*λ*). Figure 16 shows that the reflectance percentage of our calibration reference was very low. This is because of the unknown scaling factor *s,* which was used to model the more powerful direct light source compared with the diffused reflectance of the calibration target.

**Figure 18:**
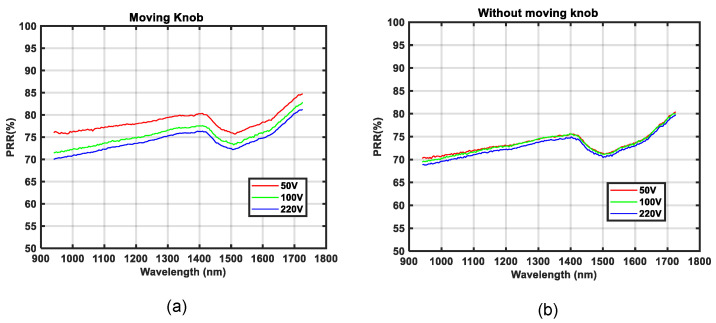
Relative reflectance *RR*(*λ*) for a brown paper as a percentage PRR(λ): (**a**) transformer knob was moved in-between measurement of the surface and reference, (**b**) the transformer knob was not moved between surface measurement and reference measurement.

As one of the purposes of this study was to measure relative reflectance independent of an illumination source, the quality of our results directly relates to how much the measurement graphs for different voltages overlap; for more details, see Figure 14. It can be seen that one of the measurements was slightly less overlapping with the other two, especially in the case of wet asphalt, as shown in Figure 14c. By investigating this result, it was found that the source of error was the limited precision of the variable transformer that was used to set the lamp voltages. To validate our findings, another experiment was conducted. Spectral measurements of brown paper at three different voltage levels: 50 V, 100 V, and 200 V, were recorded. Then the calibration reference was placed in front of the camera and the same spectral measurements were made again using lamp voltages, 50 V, 100 V, and 220 V. It was found that the results did not overlap well, as shown in Figure 18a. In a second experiment, both the paper and the calibration reference were recorded without changing the lamp voltage in-between. This experiment was performed for 50 V, 100 V, and 220 V, and it was found that all three measurements overlapped almost perfectly; see Figure 18b. From this experiment, it was concluded that the source of error must have been the precision of the lamp voltages set by the variable transformer.

The score plot of the PCA analysis of the relative reflectance, shown in Figure 15b, shows that all of the data vectors *RR*(*λ*) are grouped into distant clusters, depending on the road conditions measured. This clustering of data vectors is irrespective of the spectral distribution of light intensity of the illumination source. The large interclass and small intraclass distances emphasize that our proposed calibration method enables the classification of road conditions, even with large variations in the spectral distribution of light intensity. On the other hand, the score plot of PCA of direct reflectance *MR*(*λ*) (un-calibrated data), shown in Figure 15a, shows large intra-class and small interclass distances. Furthermore, the classification results also show that it is possible to improve the classification performance with the use of the proposed calibration method.

A calibration reference that is used in outdoor conditions must not absorb humidity such that it affects the spectral distribution of reflected light. This requirement was formulated in Section 2.1. Figure 17 shows the result of experiments conducted to determine the possible sensitivity to humidity of our PTFE calibration target reference. Measurements of *s^−1^W*(*λ*) were taken in very humid and dry conditions. As the two measurements overlap almost perfectly, this shows that the inexpensive PTFE tile chosen is close to completely insensitive to such environmental changes. It should thus be possible, e.g., to place the calibration reference on humid ground without affecting its spectral distribution of reflectance.

The empirical evidence from the experiment on road status classification is not strong, considering the limited volume on data. Only three different spectral distributions of illumination were used and only a few different locations for each class of road status were included. However, in combination with the theoretical modeling of the calibration procedure, the total evidence for the capability of presented method to suppress any impact from variable spectra of illumination *L*(*λ*) was more convincing.

The presented theoretical model comprises a complete hyperspectral imaging system, including spectral distributions of illumination, camera sensitivity, reference, and surface reflectance. The concept of dose, intensity, and exposure time was included in the model, allowing different exposure times for reference and measurements to be used. This flexible use of exposure times was incorporated into the road status experiment and also provided empirical evidence for the specific camera used. However, the normalization of intensity as modeled by Equation (4) could have become erroneous if the camera dose response curve showed more non-linearity than what was recorded for the Specim FX17 camera shown in Figure 8. If necessary, more accurate models for the dose response could have been included.

This study is lacking empirical evidence for the idea that variations in spectral distribution of camera sensitivity *C*(*λ*) can be suppressed, and only a theoretical model is provided. A reliable experiment would require the use of several very expensive cameras, preferably from different manufacturers, a cost which is not possible for the university to cover.

## 5. Conclusions

In this paper, it is shown that the relative reflectance of a surface can be measured independently of the spectral distribution of both light intensity and sensitivity of a hyperspectral camera. Measurements of relative reflectance assume the use of specific reference material during the calibration procedure. We formalized such a calibration procedure and evaluated it using a low-cost calibration reference made of PTFE. This calibration reference was confirmed to have a spectral distribution of reflectance that is independent of environmental humidity and similar to the more expensive Spectralon^TM^. Principal component analysis of relative reflectance from an asphalt surface at various light conditions further showed that the proposed calibration procedure is valid for the classification of materials.

## Figures and Tables

**Table 1:** Miss-classification rate for calibrated and uncalibrated spectral data of surfaces.

Method	Miss-Classification Rate			
	1PC	2PC	3PC	4PC
Without Calibration	75%	25.4%	25.1%	25%
With Calibration	10.5%	0%	0%	0%

## Data Availability

The data presented in this study are available on request from the corresponding author.
